# Supplementary material for: Early lineage segregation of primary myotubes from secondary myotubes and adult muscle stem cells
Source: Nat Commun. 2025 Aug 22;16:7858. doi: 10.1038/s41467-025-61767-1 (PMC12374003; doi:10.1038/s41467-025-61767-1)
Supplement: Supplementary file 2 — Description of Additional Supplementary Information [file 41467_2025_61767_MOESM2_ESM.docx]

**Description of Additional Supplementary Information**

***Supplementary Data 1****:*

**Differentially expressed genes for each myogenic clusters. C**ontains the list of differentially expressed genes (DEGs) identified per cluster in the single-cell RNA-seq dataset, including gene name, log₂ fold change (log2FC), average expression, adjusted p-value (FDR), and cluster identity for each gene.

***Supplementary Data 2:***

**Differentially expressed genes in between the 16TF-VNP+ and the 16TF-VNP- populations.**Contains the list of differentially expressed genes (DEGs) resulting from a comparison between the 16TF-VNP+ and16TF-VNP- population. It includes gene names, log fold change (log2FC), average expression and p-value
