# Supplementary material for: Early lineage segregation of primary myotubes from secondary myotubes and adult muscle stem cells
Source: Nat Commun. 2025 Aug 22;16:7858. doi: 10.1038/s41467-025-61767-1 (PMC12374003; doi:10.1038/s41467-025-61767-1)
Supplement: Supplementary file 5 — Reporting Summary [file 41467_2025_61767_MOESM5_ESM.pdf]

nature portfolio

Corresponding author(s): Christophe Marcelle

Last updated by author(s): 2025/04/04

Reporting Summary

Nature Portfolio wishes to improve the reproducibility of the work that we publish. This form provides structure for consistency and transparency in reporting. For further information on Nature Portfolio policies, see our Editorial Policies and the Editorial Policy Checklist.

Statistics

For all statistical analyses, confirm that the following items are present in the figure legend, table legend, main text, or Methods section.

n/a	Confirmed	
☐	☒	The exact sample size (*n*) for each experimental group/condition, given as a discrete number and unit of measurement
☒	☐	A statement on whether measurements were taken from distinct samples or whether the same sample was measured repeatedly
☐	☒	The statistical test(s) used AND whether they are one- or two-sided<br>*Only common tests should be described solely by name; describe more complex techniques in the Methods section.*
☒	☐	A description of all covariates tested
☐	☒	A description of any assumptions or corrections, such as tests of normality and adjustment for multiple comparisons
☐	☒	A full description of the statistical parameters including central tendency (e.g. means) or other basic estimates (e.g. regression coefficient) AND variation (e.g. standard deviation) or associated estimates of uncertainty (e.g. confidence intervals)
☐	☒	For null hypothesis testing, the test statistic (e.g. *F*, *t*, *r*) with confidence intervals, effect sizes, degrees of freedom and *P* value noted<br>*Give P values as exact values whenever suitable.*
☒	☐	For Bayesian analysis, information on the choice of priors and Markov chain Monte Carlo settings
☒	☐	For hierarchical and complex designs, identification of the appropriate level for tests and full reporting of outcomes
☒	☐	Estimates of effect sizes (e.g. Cohen's *d*, Pearson's *r*), indicating how they were calculated

Our web collection on statistics for biologists contains articles on many of the points above.

Software and code

Policy information about availability of computer code

Data collection	BD FACSDIVA software (v9.0.1)
Data analysis	fastqc, fastq_screen, STAR (v2.7.9a), Ensembl (GRCg6a.105), Seurat (v5.1.0), glmGamPoi, scDblFinder (v1.16.0), igraph, FItSNE, MAST, R (v4.4.3), python (v3.10.16), scanpy (v1.10.4), numpy (v2.0.2), matplotlib (v3.10.0), igraph (v0.11.8), Fiji (v2.7.0)

For manuscripts utilizing custom algorithms or software that are central to the research but not yet described in published literature, software must be made available to editors and reviewers. We strongly encourage code deposition in a community repository (e.g. GitHub). See the Nature Portfolio guidelines for submitting code & software for further information.

Data

Policy information about availability of data

All manuscripts must include a data availability statement. This statement should provide the following information, where applicable:

- Accession codes, unique identifiers, or web links for publicly available datasets
- A description of any restrictions on data availability
- For clinical datasets or third party data, please ensure that the statement adheres to our policy

scRNA-sequencing data sets for chicken embryos are available at the Gene Expression Omnibys (GEO) under the accession number GSE283478.

Research involving human participants, their data, or biological material

Policy information about studies with human participants or human data. See also policy information about sex, gender (identity/presentation), and sexual orientation and race, ethnicity and racism.

Reporting on sex and gender	Human data analyzed in this study was already publised in Zhang et al. 2023 (https://doi.org/10.1038/s41586-023-06806-x) and are publicly accessible.
Reporting on race, ethnicity, or other socially relevant groupings	See Zhang et al. 2023
Population characteristics	See Zhang et al. 2023
Recruitment	See Zhang et al. 2023
Ethics oversight	See Zhang et al. 2023

Note that full information on the approval of the study protocol must also be provided in the manuscript.

Field-specific reporting

Please select the one below that is the best fit for your research. If you are not sure, read the appropriate sections before making your selection.

- [x] Life sciences
- [ ] Behavioural & social sciences
- [ ] Ecological, evolutionary & environmental sciences

For a reference copy of the document with all sections, see nature.com/documents/nr-reporting-summary-flat.pdf

Life sciences study design

All studies must disclose on these points even when the disclosure is negative.

Sample size	For experiments that were analyzed in wholemount, each dot represents an embryo. For cryosections, each dot represents a section, and the total number of embryos is indicated in the figure legend. For the Cxcr4 experiment, each dot represents a cell, and the total number of embryos is indicated in the figure legend. For each condition, a minimum of 4 embryos were analyzed and for each embryo or section, a minimum of 250 cells were counted, leading to a total number of 1000 to 10 000 cells per condition. Experiments with two different conditions were compared using Wilcoxon-Mann-Whitney test and experiment with three different conditions with Kruskal-Wallis test associated with Dunn-Bonferroni post-hoc test. NS represent a p-value>0.05, * a p-value<0.01, *** a p-value<0.001 and **** a p-value<0.0001. for the lineage tracing experiments, a mean of 7 embryo was used per condition. Chicken embryo, as any other amniotes, exhibit a very stereotypical development. We used a total number of 4 to 12 chicken embryos for each condition or experiment, as this sample size is sufficient to capture the typical developmental variability observed in chicken embryos. Chicken embryos exhibit a highly regulated and consistent pattern of development, which allows for meaningful conclusions to be drawn from this range of embryos. This sample size was based on several of our previous studies (https://doi.org/10.1038/s41467-020-20290-1 ; DOI: 10.1038/nature09970).
Data exclusions	No data was excluded
Replication	Each electroporation, grafting and anlaysis was done the same way and only by GT to ensure comparable outputs. Each electroporation results comes from at least two independant experiments.
Randomization	Embryos were asigned randomly in each experimental group
Blinding	We did not analyze our data using a double-blind approach because the study involved observing a population of cells without comparing two distinct experimental conditions. Since the primary goal was to examine the natural behavior of the cells within a single condition, blinding was not necessary for the analysis. The focus was on descriptive observations rather than experimental comparisons that would require such controls. For the DNLEF1 experiment, the migratory phenotype was so pronounced that double-blinding would not have been feasible or meaningful, as the effects were highly visible and easily distinguishable. In the case of Cxcr4 quantification, the DNLEF1-expressing nuclei were marked by dTomato, making them easily recognizable and eliminating the pertinence of blinding. Regarding the grafts experiment, the two conditions were analyzed the same way but without blinding.

Reporting for specific materials, systems and methods

We require information from authors about some types of materials, experimental systems and methods used in many studies. Here, indicate whether each material, system or method listed is relevant to your study. If you are not sure if a list item applies to your research, read the appropriate section before selecting a response.

Materials & experimental systems

n/a	Involved in the study	
☐	☒	Antibodies
☐	☒	Eukaryotic cell lines
☒	☐	Palaeontology and archaeology
☐	☒	Animals and other organisms
☒	☐	Clinical data
☒	☐	Dual use research of concern
☒	☐	Plants

Methods

n/a	Involved in the study	
☒	☐	ChIP-seq
☐	☒	Flow cytometry
☒	☐	MRI-based neuroimaging

Antibodies

Antibodies used

Rabbit polyclonal antibody (IgG) against dTomato
Concentration: 1/1000
Supplier: Abcam
Reference: ab62341

Chicken polyclonal antibody (IgY) against eGFP, mVenus, and Achilles
Concentration: 1/1000
Supplier: Invitrogen
Reference: A10262

Mouse monoclonal antibody (IgG2a) against eGFP, mVenus, and Achilles
Concentration: 1/1000
Supplier: Invitrogen
Reference: A11120

Rabbit polyclonal antibody (IgG) against TagBFP
Concentration: 1/500
Supplier: Evrogen
Reference: AB233

Mouse monoclonal antibody (IgG1) against PAX7
Concentration: 1/10
Supplier: DSHB
Reference: AB528428
Clone Name: PAX7

Mouse monoclonal antibody (IgG2b) against Myosin Heavy Chain (MF20)
Concentration: 1/10
Supplier: DSHB
Reference: AB2147781
Clone Name: MF20

Mouse monoclonal antibody (IgA) against the slow myosin MYH7B (S58)
Concentration: 1/10
Supplier: DSHB
Reference: AB528377
Clone Name: S58

Alexa Fluor® 488 conjugated Goat anti-Rabbit IgG (H+L)
Supplier: ThermoFisher
Reference: A11008

Alexa Fluor® 488 conjugated Goat anti-Mouse IgG1 (H+L)
Supplier: ThermoFisher
Reference: A21121

Alexa Fluor® 488 conjugated Goat anti-Mouse IgG2a (H+L)
Supplier: ThermoFisher
Reference: A21131

Alexa Fluor® 488 conjugated Goat anti-Mouse IgG2b (H+L)
Supplier: ThermoFisher

Reference: A21141

Alexa Fluor® 555 conjugated Goat anti-Rabbit IgG (H+L)
Supplier: ThermoFisher
Reference: A21428

Alexa Fluor® 555 conjugated Goat anti-Mouse IgG1 (H+L)
Supplier: ThermoFisher
Reference: A21421

Alexa Fluor® 555 conjugated Goat anti-Mouse IgG2a (H+L)
Supplier: ThermoFisher
Reference: A21423

Alexa Fluor® 555 conjugated Goat anti-Mouse IgG2b (H+L)
Supplier: ThermoFisher
Reference: A21425

Alexa Fluor® 647 conjugated Goat anti-Rabbit IgG (H+L)
Supplier: ThermoFisher
Reference: A21245

Alexa Fluor® 647 conjugated Goat anti-Mouse IgG1 (H+L)
Supplier: ThermoFisher

Alexa Fluor® 647 conjugated Goat anti-Mouse IgG2a (H+L)
Supplier: ThermoFisher
Reference: A21241

Alexa Fluor® 647 conjugated Goat anti-Mouse IgG2b (H+L)
Supplier: ThermoFisher
Reference: A21251

Validation

All the primary antibodies targeting fluorescent proteins were tested by immunostaining electroporated samples with secondaries antibodies fluorescing in a different wavelenght.

Eukaryotic cell lines

Policy information about cell lines and Sex and Gender in Research

Cell line source(s)	HEK-293
Authentication	Cells were purchased directly from ATCC ((#CRL-1573, ATCC). When used our cells exhibited the typical HEK293 morphology
Mycoplasma contamination	Our cell line of HEK293 was negative for mycoplasma
Commonly misidentified lines (See ICLAC register)	*Name any commonly misidentified cell lines used in the study and provide a rationale for their use.*

Animals and other research organisms

Policy information about studies involving animals; ARRIVE guidelines recommended for reporting animal research, and Sex and Gender in Research

Laboratory animals	Fertilized eggs from a local breeder (naked neck breed)
Wild animals	No wild animals where used in this study
Reporting on sex	The sex of the embryo was not taked into account
Field-collected samples	No field collection
Ethics oversight	No ethics was necessary for work on chicken embryos

Note that full information on the approval of the study protocol must also be provided in the manuscript.

Plants

Seed stocks	N/A
Novel plant genotypes	N/A
Authentication	N/A

Flow Cytometry

Plots

Confirm that:

- [x] The axis labels state the marker and fluorochrome used (e.g. CD4-FITC).
- [x] The axis scales are clearly visible. Include numbers along axes only for bottom left plot of group (a 'group' is an analysis of identical markers).
- [x] All plots are contour plots with outliers or pseudocolor plots.
- [x] A numerical value for number of cells or percentage (with statistics) is provided.

Methodology

Sample preparation	E4.5 electroporated chicken embryos with a ubiquitous dTomato and the 16TF-VNP reporter were screen under a fluorescent binocular and electroporated limb buds were quickly dissected and incubated with 500 µl of pre-warmed Dispase (1,5mg/ml in DMEM / 10mM Hepes), pipette up and down 10 times and incubated 15min at 37°C. The sample was homogenized every 5min then 500 µl of pre-warmed Trypsin (0,05% in DMEM) was added to the tube, homogenized and incubated 3min at 37°C. Samples were then transferred into a 15ml falcon tube, and the reaction was stop with 10ml of Hanks buffer (for 100ml: 10ml of HBSS 10X, 250mg of BSA, 1ml of Hepes 1M, in sterile ddH2O), homogenized and centrifugated 10min at 500g. The pellet was re-suspended in 4ml of Hanks buffer and filtered with a pre-humidified 40 µm sterile filter and re-centrifugated 10min at 500g. The final pellet was re-suspended into 250 µl of Hanks buffer and added to 250 µl of Hanks buffer in a pre-humidified FACS tube. For sorting, we added DAPI (1/1000) in the final Hanks buffer solution and prepare a sample containing non-electroporated tissues and non-electroporated tissues stained with DAPI to calibrate the sorting. Cells were then sorting according to the dTomato fluorescence and collected into Hank's buffer. An example of the fluorescence-activated cell sorting (FACS) gating strategy is provided in Fig. S12. For the single-cell RNA-seq experiment, a total of 6 electroporated limb buds were pooled together in the same tube.
Instrument	BD FACSDiva 9.0.1
Software	BD FACSDiva Software
Cell population abundance	0,8% of the total events, 1,2% of all lived cells
Gating strategy	The gating strategy for sorting tdTomato+ cells involved first excluding debris and doublets by plotting FSC-A (forward scatter area) against SSC-A (side scatter area), and selecting the main cell population. Next, a gate was applied to exclude doublets by plotting FSC-H (forward scatter height) versus FSC-A. Finally, tdTomato+ cells were identified by gating on the fluorescence intensity corresponding to the tdTomato signal, ensuring that only the target cells were sorted.

- [x] Tick this box to confirm that a figure exemplifying the gating strategy is provided in the Supplementary Information.
