# Supplementary material for: Early lineage segregation of primary myotubes from secondary myotubes and adult muscle stem cells
Source: Nat Commun. 2025 Aug 22;16:7858. doi: 10.1038/s41467-025-61767-1 (PMC12374003; doi:10.1038/s41467-025-61767-1)
Supplement: Supplementary file 1 — Supplementary Information [file 41467_2025_61767_MOESM1_ESM.pdf]

Early lineage segregation of primary myotubes from secondary myotubes and adult muscle stem cells

Gauthier Toulouse[1], William Jarassier[2,§], Sabrina Jagot[1,§],Valérie Morin[1], Fabien Le Grand[1,2,*], Christophe Marcelle[1,3,*]

1: Université Claude Bernard Lyon1, MeLiS Laboratory, CNRS, INSERM, Lyon, France
2: Université Claude Bernard Lyon1, PGNM Laboratory, CNRS, INSERM, Lyon, France
3: Monash University, Australian Regenerative Medicine Institute, Clayton, Victoria, Australia
**: Corresponding authors*
[§]: these authors contributed equally

Supplementary information

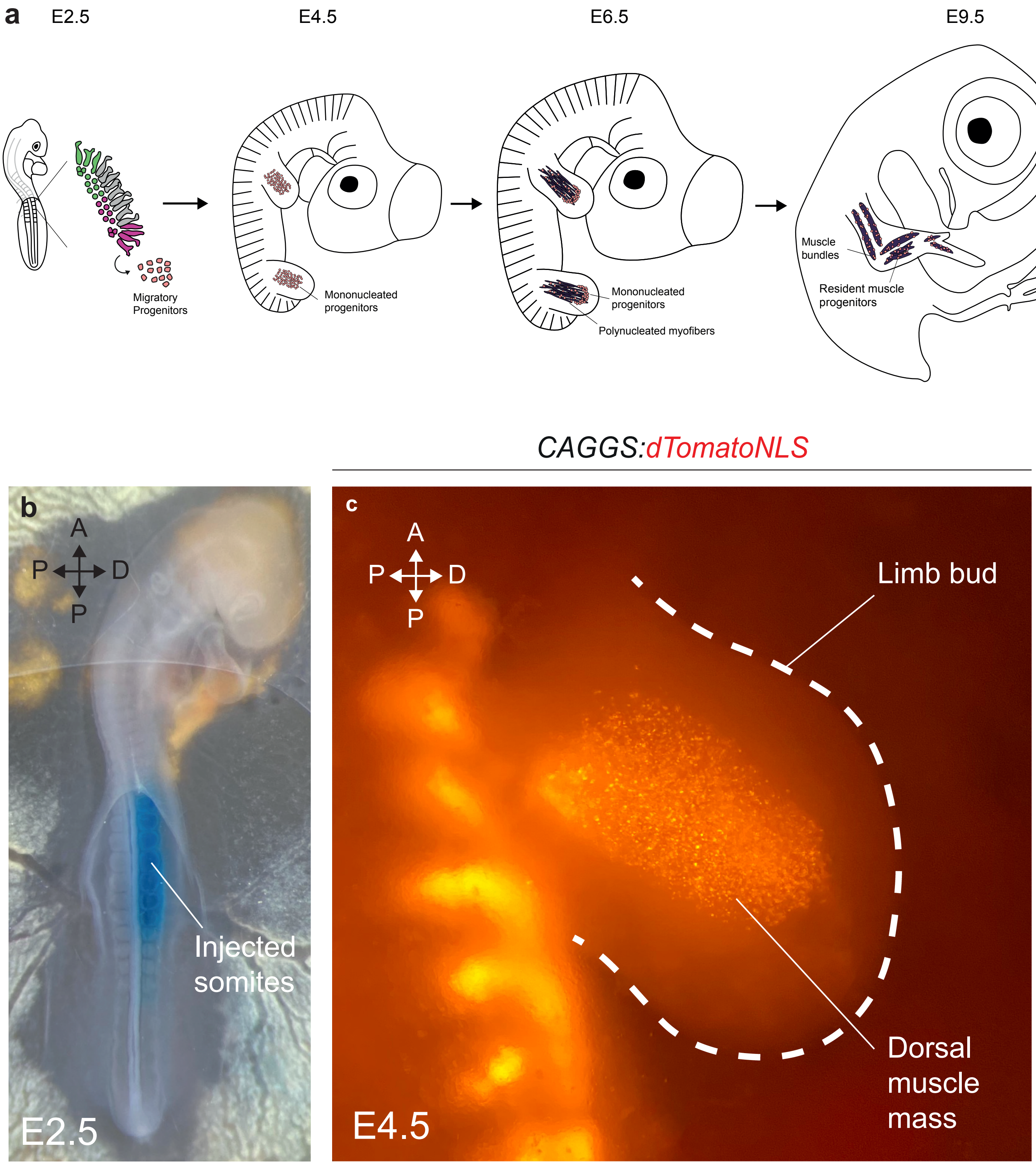
a
E2.5
E4.5
E6.5
E9.5
Migratory Progenitors
Mononucleated progenitors
Mononucleated progenitors
Polynucleated myofibers
Muscle bundles
Resident muscle progenitors
CAGGS:dTomatoNLS
b
A
P
D
P
Injected somites
E2.5
c
A
P
D
P
Limb bud
Dorsal muscle mass
E4.5

Supplementary Figure 1: Brachial somites electroporation. a Between E2.5 and E4.5, epithelial cells from the ventro-lateral lip of dermomyotomes 16–21 delaminate and migrate distally to invade the growing limb bud. By E4.5, they form a pool of mononucleated muscle progenitors within the limb bud. These progenitors are organized into a dorsal and ventral muscle masses (not shown). At this stage, all of them robustly express *Myf5*, while *MyoD* expression is only beginning. Two days later, at E6.5, some muscle progenitors have elongated and fused to form multinucleated myofibers, which remain intermingled with mononucleated progenitors. By E9.5, the two initial muscle masses have segregated into distinct muscle bundles, reflecting the adult organization. Each muscle bundle is composed of primary myotubes and contains resident muscle progenitors. **b** At E2.5 (HH15), a plasmid mix is injected into the cavity of epithelial somites 16–21, and a current is applied to target the lateral part of the somites. **c** Macroscopic view of a typical limb at E4.5 invaded by electroporated muscle progenitors; the border of the limb bud is delineated with dotted lines.

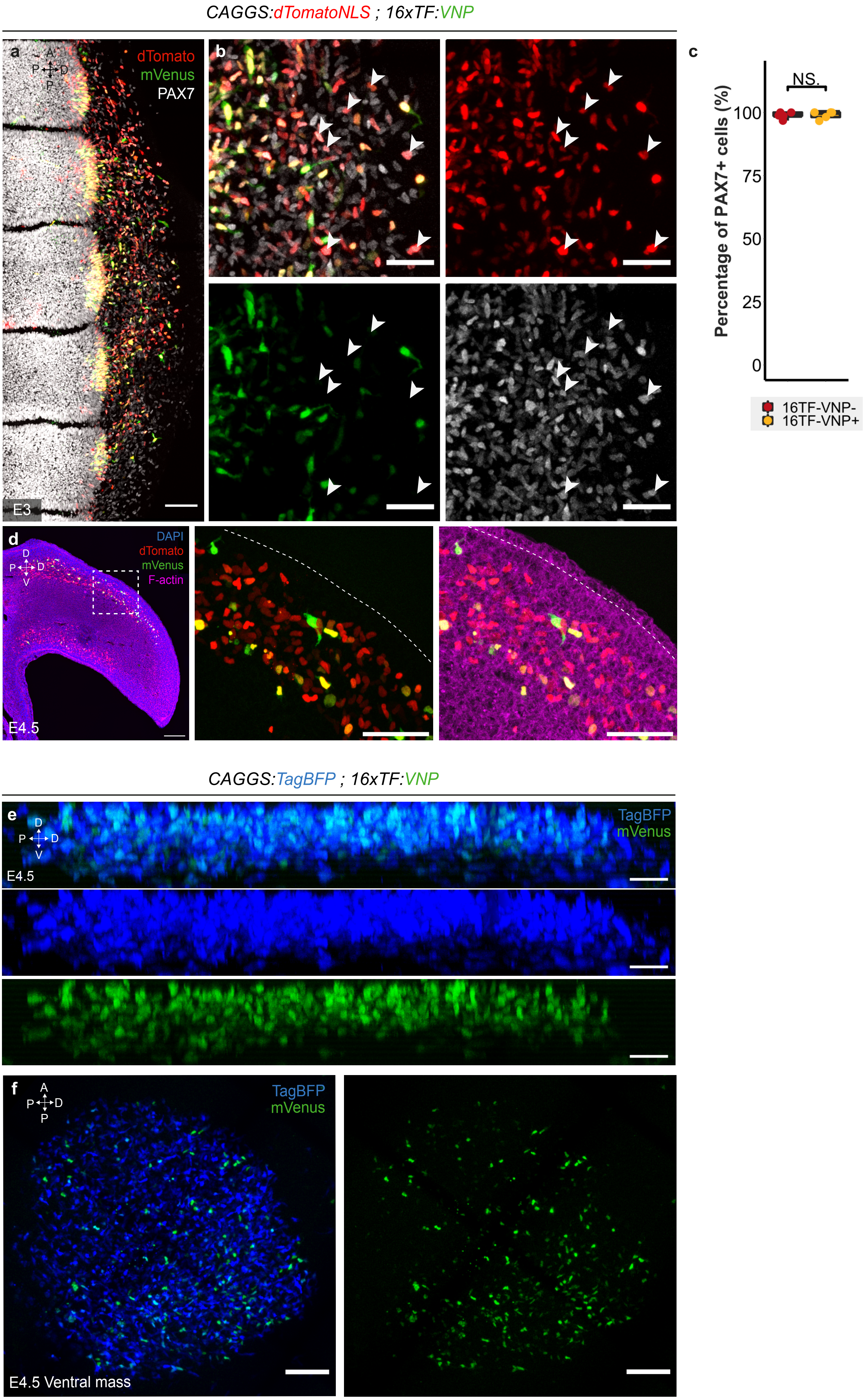

CAGGS:dTomatoNLS ; 16xTF:VNP
a
dTomato
mVenus
PAX7
E3
b
c
NS.
Percentage of PAX7+ cells (%)
100
75
50
25
0
16TF-VNP-
16TF-VNP+
d
DAPI
dTomato
mVenus
F-actin
E4.5
CAGGS:TagBFP ; 16xTF:VNP
e
TagBFP
mVenus
E4.5
f
TagBFP
mVenus
E4.5 Ventral mass

Supplementary Figure 2: Early expression of the 16TF-VNP reporter in muscle progenitors. **a,b** Dorsal view of a confocal stack of E3 brachial somites electroporated with a ubiquitous dTomato, the 16TF-VNP reporter and stained for PAX7. (**b**) is an enlargement of (**a**), arrowheads indicate electroporated cells that are 16TF-VNP^{-} and positive for PAX7. **c** Quantification of the percentage of PAX7^{+} cells that are 16TF-VNP^{-} and 16TF-VNP^{+}, each dot represents an embryo, n=5. Statistical comparison was performed using the two-sided non-parametric Wilcoxon test. NS: p>0,05. **d** Longitudinal section of E4.5 limb electroporated with a ubiquitous dTomato and the 16TF-VNP reporter. Dotted line indicates the junction between the dermis and the epidermis. **e** Longitudinal optical section of limb dorsal muscle mass electroporated with a ubiquitous TagBFP and the 16TF-VNP reporter. **f** Ventral view of a confocal stack of the ventral muscle mass of a E4.5 limb bud electroporated with a ubiquitous TagBFP and the 16TF-VNP reporter (n=4 embryos). Box plots show the median (center line), 25th and 75th percentiles (box limits), and whiskers extending to the smallest and largest values within 1.5xIQR. Scale bars: 50µm (**b**), or 100µm (**a, d-f**)

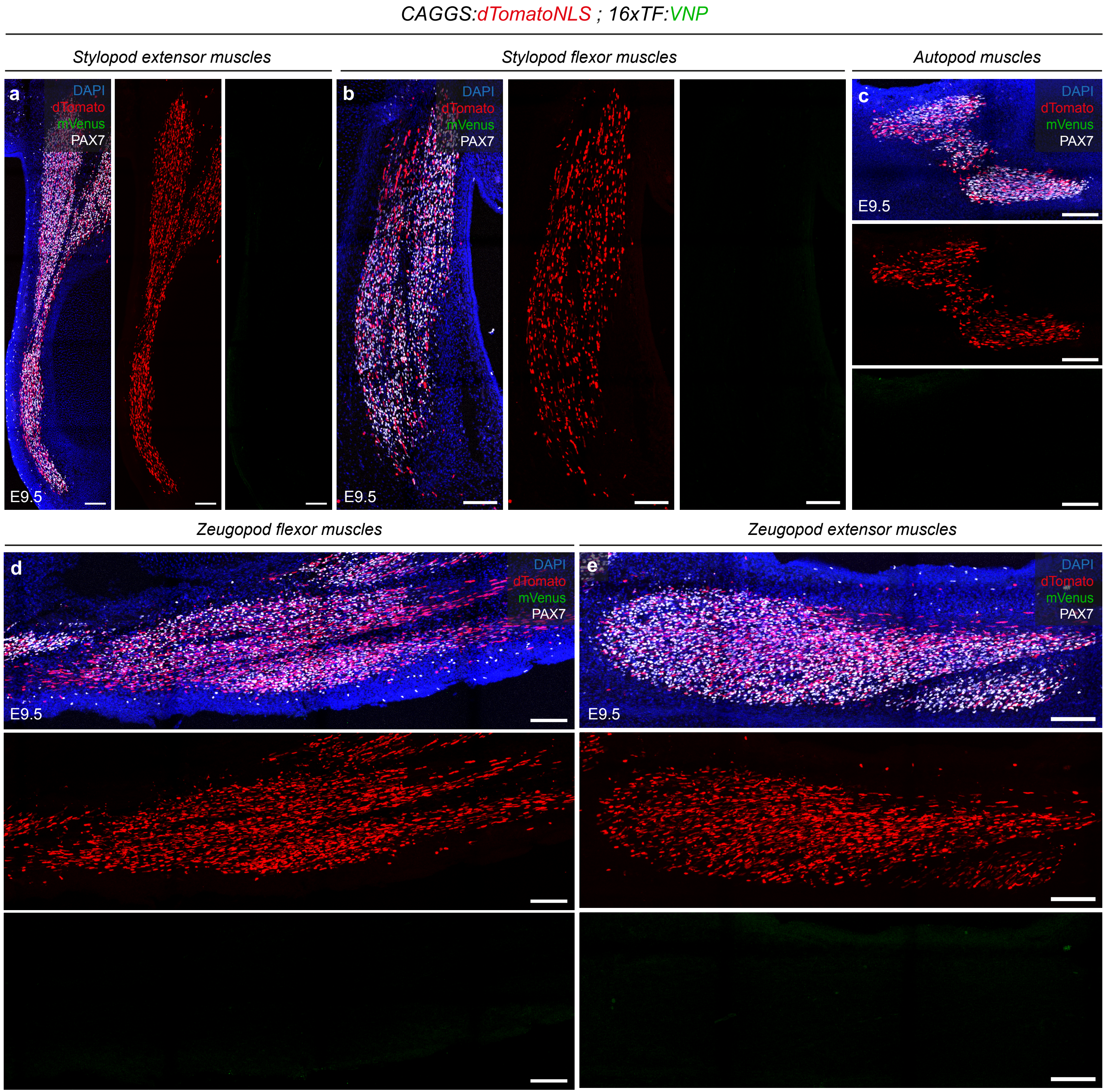
CAGGS:dTomatoNLS ; 16xTF:VNP
Stylopod extensor muscles
Stylopod flexor muscles
Autopod muscles
a
b
c
DAPI
dTomato
mVenus
PAX7
E9.5
Zeugopod flexor muscles
Zeugopod extensor muscles
d
e

Supplementary Figure 3: The 16TF-VNP reporter is not active in late embryonic muscle masses. **a-e** Longitudinal sections of E9.5 limb electroporated with a ubiquitous nuclear dTomato, the 16TF-VNP reporter and immunostained for PAX7 at various anatomical locations along the limb proximo-distal axis, n=8 embryos. Scale bars: 100µm (**a-e**)

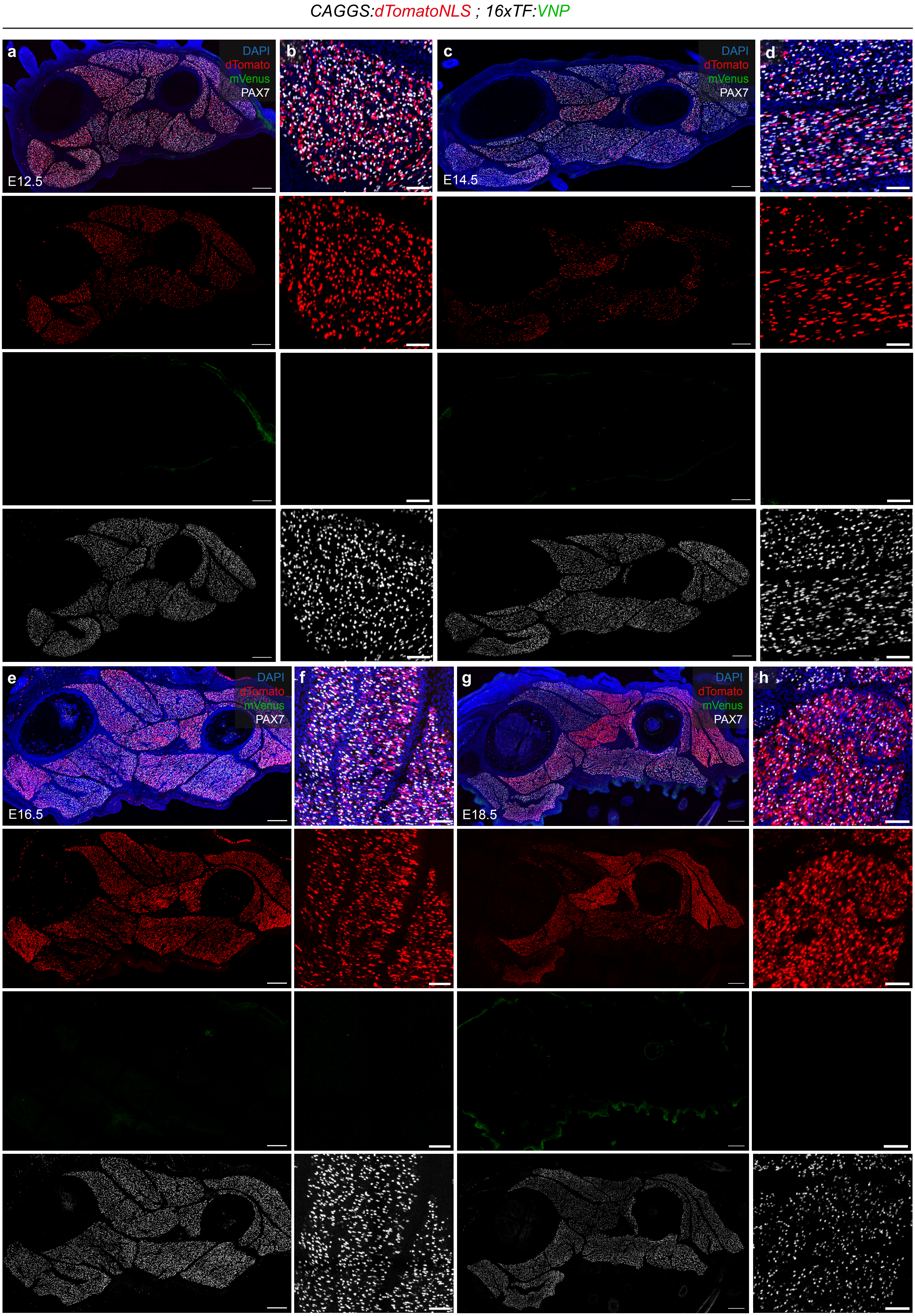
CAGGS:dTomatoNLS ; 16xTF:VNP
a
DAPI
dTomato
mVenus
PAX7
E12.5
b
c
E14.5
d
e
E16.5
f
g
E18.5
h

Supplementary Figure 4: The 16TF-VNP reporter is not active in muscle masses in fetal stages. **a-h** Transversal sections of E12.5 (**a**,**b**), E14.5 (**c**,**d**), E16.5 (**e**,**f**) and E18.5 (**g**,**h**) limb muscles electroporated with a ubiquitous nuclear dTomato, the 16TF-VNP reporter and immunostained for PAX7. (**b**), (**d**), (**f**), (**h**) are enlargements of (**a**), (**c**), (**e**), (**g**) respectively, n=6, 6, 4 and 4 limbs for each stage, respectively. Scale bars: 50µm.

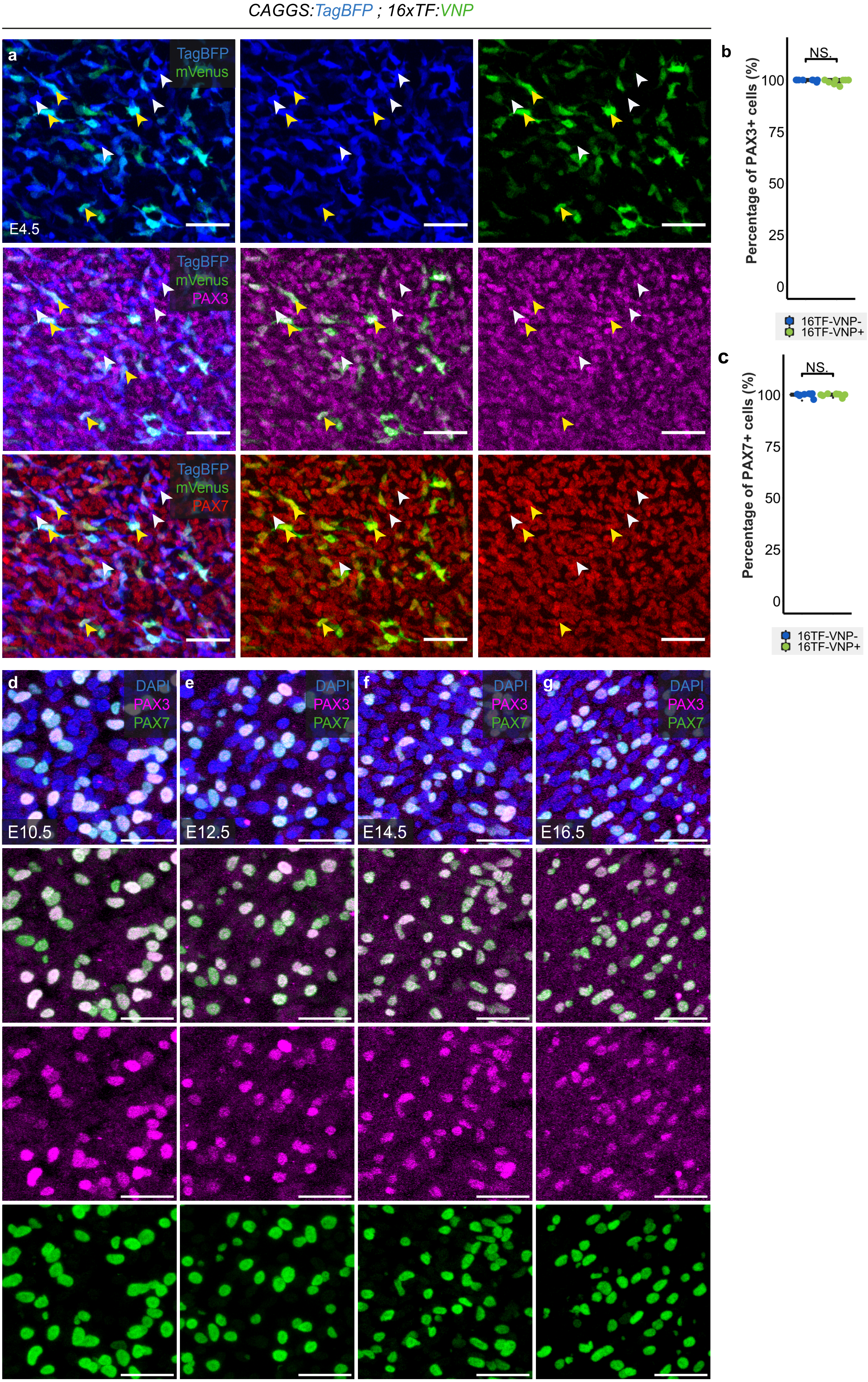

CAGGS:TagBFP ; 16xTF:VNP
a
TagBFP
mVenus
E4.5
TagBFP
mVenus
PAX3
TagBFP
mVenus
PAX7
b
NS.
100
75
50
25
0
Percentage of PAX3+ cells (%)
16TF-VNP-
16TF-VNP+
c
NS.
100
75
50
25
0
Percentage of PAX7+ cells (%)
16TF-VNP-
16TF-VNP+
d
e
f
g
DAPI
PAX3
PAX7
E10.5
E12.5
E14.5
E16.5

Supplementary Figure 5: *Pax3* and *Pax7* expression patterns during myogenesis. **a** Dorsal view of a confocal stack of a E4.5 limb bud electroporated with a ubiquitous TagBFP, the 16TF-VNP reporter and immunostained for PAX3 and PAX7. White arrowheads indicate 16TF-VNP$^-$ cells, yellow arrowheads indicate 16TF-VNP$^+$ cells. **b** Quantification of the percentage of PAX3$^+$ cells within the 16TF-VNP$^-$ and 16TF-VNP$^+$ populations, each dot represents a limb bud, n=9. Statistical comparison was performed using the two-sided non-parametric Wilcoxon test. NS: $p>0{,}05$. **c** Quantification of the percentage of PAX7$^+$ cells within the 16TF-VNP$^-$ and 16TF-VNP$^+$ populations, n=9. **d-g** Transversal sections of limb muscles from E10.5 (**e**), E12.5 (**f**), E14.5 (**g**) and E16.5 (**h**) fetuses and immunostained for PAX3 and PAX7, n=2 for each stage. Box plots show the median (center line), 25th and 75th percentiles (box limits), and whiskers extending to the smallest and largest values within 1.5xIQR. Scale bars: 50µm.

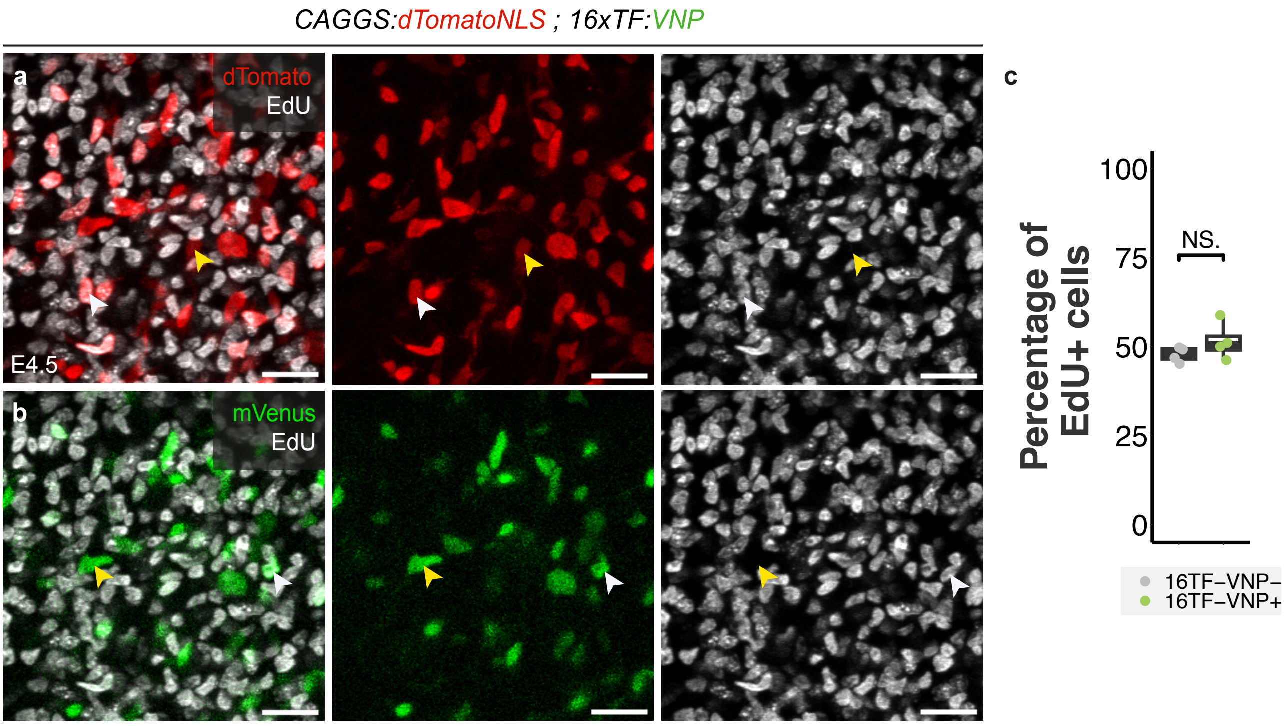

CAGGS:dTomatoNLS ; 16xTF:VNP
a
dTomato
EdU
E4.5
b
mVenus
EdU
c
Percentage of EdU+ cells
100
75
50
25
0
NS.
16TF–VNP–
16TF–VNP+

Supplementary Figure 6: 16TF-VNP$^+$ and 16TF-VNP$^-$ progenitors proliferate at the same rate. **a-b** Dorsal view of a confocal stack of E4.5 limb buds electroporated with a ubiquitous nuclear dTomato, the 16TF-VNP reporter and labeled with EdU to detect proliferating cells. White arrowheads indicate EdU$^+$ cells; yellow arrowhead indicates EdU$^-$ cells. **c** Quantification of EdU$^+$/16TF-VNP$^+$ and EdU$^+$/16TF-VNP$^-$ progenitors. Each dot represents a limb bud, n=4. Statistical comparison was performed using the two-sided non-parametric Wilcoxon test. NS: $p>0,05$. Box plots show the median (center line), 25th and 75th percentiles (box limits), and whiskers extending to the smallest and largest values within 1.5xIQR. Scale bars: 50µm

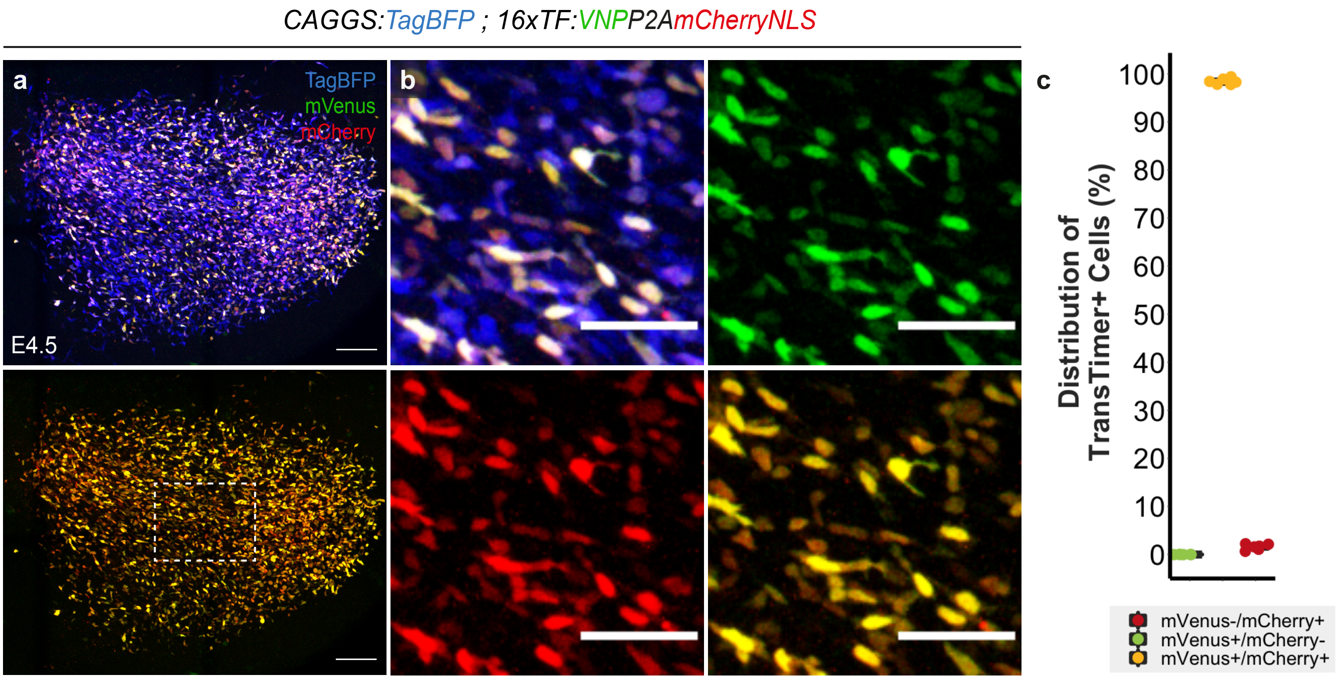

CAGGS:TagBFP ; 16xTF:VNPP2AmCherryNLS
a
TagBFP
mVenus
mCherry
E4.5
b
c
Distribution of TransTimer+ Cells (%)
100
90
80
70
60
50
40
30
20
10
0
mVenus-/mCherry+
mVenus+/mCherry-
mVenus+/mCherry+

Supplementary Figure 7: Pseudo-lineage of 16TF-VNP+ cells. a,**b** Dorsal view of a confocal stack of E4.5 limb bud electroporated with a ubiquitous TagBFP and the 16TF-VNP-P2A-mCherryNLS construct. (**a**) is an enlargement of (**b**). **c** Quantification (in percentage) of electroporated progenitors positive or negative for the mVenus and the mCherry. Each dot represents a limb bud, n=6. Box plots show the median (center line), 25th and 75th percentiles (box limits), and whiskers extending to the smallest and largest values within 1.5xIQR. Scale bars: 50µm (**b**) or 100µm (**a**)

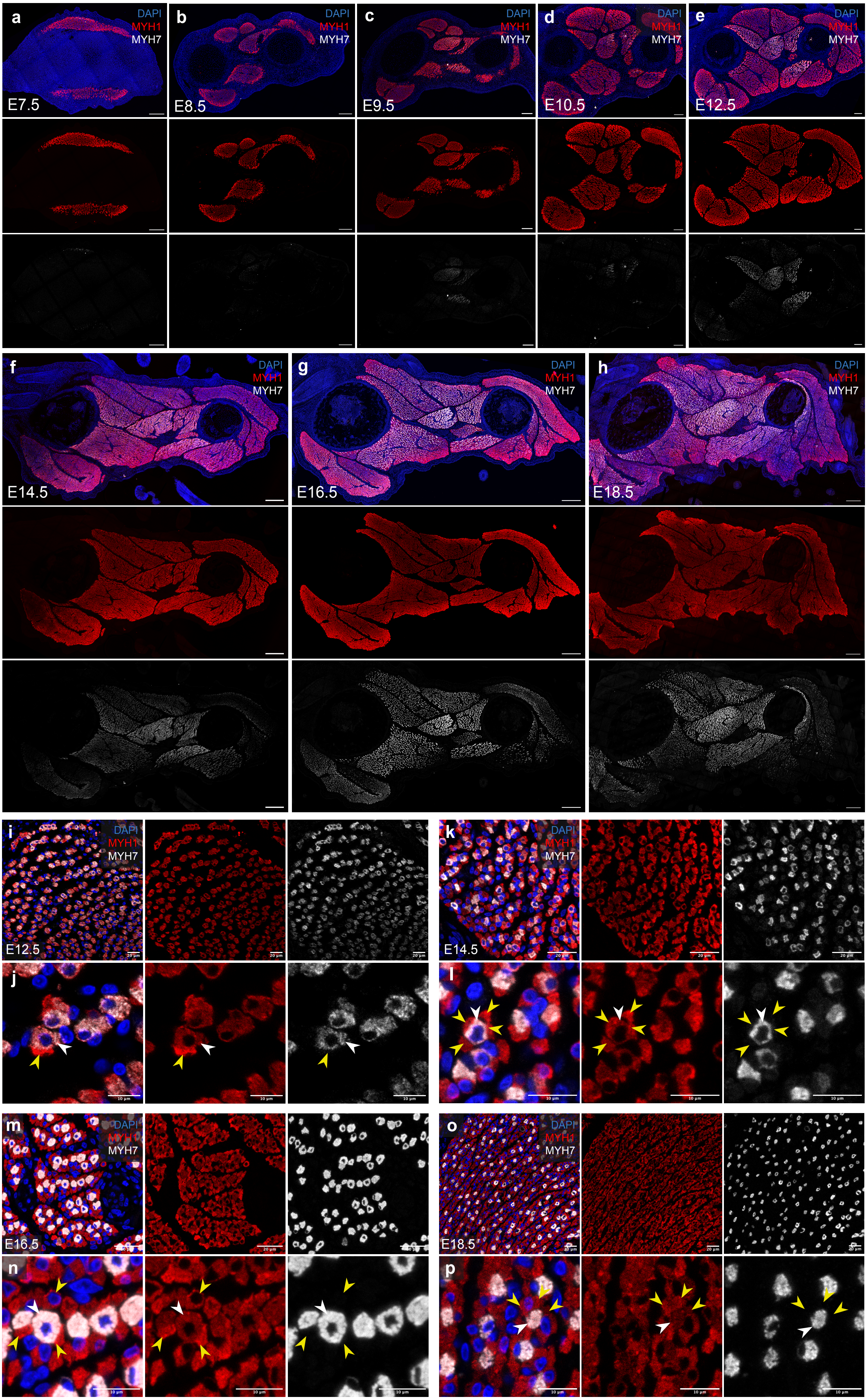

a
b
c
d
e
DAPI
MYH1
MYH7
E7.5
E8.5
E9.5
E10.5
E12.5
f
g
h
E14.5
E16.5
E18.5
i
j
k
l
m
n
o
p
E12.5
E14.5
E16.5
E18.5

Supplementary Figure 8: Expression of slow myosin throughout embryonic and fetal limb bud development. a-h Transversal sections of whole limb from E7.5 to E18.5 immunostained with the MF20 antibody labels sarcomeric myosin heavy chain (MyHC) isoforms expressed in skeletal and cardiac muscle and the S58 antibody that specifically recognize MYH7. **i-p** Enlargements of limb muscles from E12.5 to E18.5 showing primary myotubes ($MYH7^{+}$, white arrowheads) surrounded by smaller presumably secondary myotubes ($MYH7^{-}$, yellow arrowheads). n=3 limbs for each stage. Scale bars: 10µm (**j**,**l**,**n**,**p**), 20µm (**i**,**k**,**m**,**o**) or 100µm (**a**-**h**).

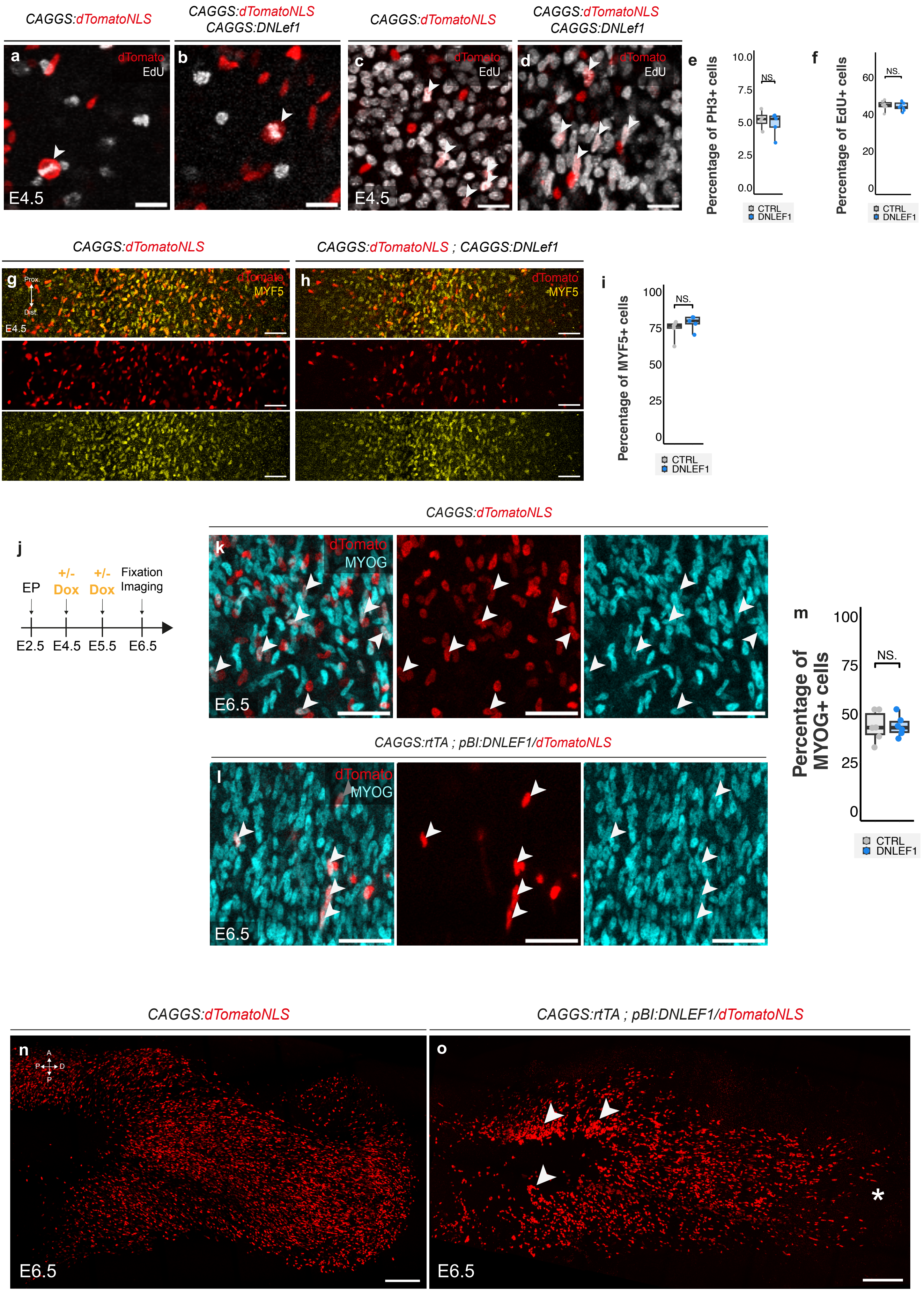
CAGGS:dTomatoNLS
CAGGS:dTomatoNLS CAGGS:DNLef1
CAGGS:dTomatoNLS
CAGGS:dTomatoNLS CAGGS:DNLef1
a
dTomato
EdU
E4.5
b
c
dTomato
EdU
E4.5
d
dTomato
EdU
e
Percentage of PH3+ cells
10.0
7.5
5.0
2.5
0.0
NS.
CTRL
DNLEF1
f
Percentage of EdU+ cells
60
40
20
0
NS.
CTRL
DNLEF1
CAGGS:dTomatoNLS
CAGGS:dTomatoNLS ; CAGGS:DNLef1
g
Prox.
Dist.
E4.5
dTomato
MYF5
h
dTomato
MYF5
i
Percentage of MYF5+ cells
100
75
50
25
0
NS.
CTRL
DNLEF1
j
EP
+/- Dox
+/- Dox
Fixation Imaging
E2.5
E4.5
E5.5
E6.5
CAGGS:dTomatoNLS
k
dTomato
MYOG
E6.5
CAGGS:rtTA ; pBI:DNLEF1/dTomatoNLS
l
dTomato
MYOG
E6.5
m
Percentage of MYOG+ cells
100
75
50
25
0
NS.
CTRL
DNLEF1
CAGGS:dTomatoNLS
CAGGS:rtTA ; pBI:DNLEF1/dTomatoNLS
n
A
P
D
P
E6.5
o
E6.5
*

Supplementary Figure 9: Inhibition of TCF-LEF/β-catenin does not affect myoblast proliferation or myogenic differentiation but regulates their spatial distribution. a-d Dorsal view of a confocal stack of E4.5 limb buds electroporated with a ubiquitous nuclear dTomato, alone (**a**,**c**) or together with a ubiquitously expressed DN Lef1 (**b**,**d**), stained for Phospho-histone H3 (**a**,**b**) or EdU (**c**,**d**), arrowheads indicate $dTomato^{+}/PH3^{+}$ or $dTomato^{+}/EdU^{+}$ cells. **e**,**f** are quantifications of (**a-d**), each dot represents a limb bud, n=4 and 5 for (e), n=7 and 9 for (f). **g**,**h** Dorsal view of a confocal stack of E4.5 limb buds electroporated with by ubiquitous nuclear dTomato (in red) alone (**g**), or together with a ubiquitous DN Lef1 (**h**) and stained for MYF5. **i** is a quantification of (**g**,**h**) each dot represents a limb bud, n=5 in both conditions. **j** depicts the experimental protocol used in (**k-o**) where DN Lef1 expression is activated by addition of doxycycline from E4.5. **k**,**l** are close-up of confocal stacks of E6.5 limb buds electroporated with a ubiquitous nuclear dTomato alone (**k**), or with a ubiquitous rtTA and a plasmid expressing both a nuclear dTomato and the DN Lef1 upon doxycycline induction (**l**) and immunostained for MYOG. Arrowheads indicate $dTomato^{+}/MYOG^{+}$ nuclei. **m** is a quantification of (**k**,**l**) each dot represents a limb bud, n=6 for each condition. **n**,**o** are full field of view of (**k**) and (**l**), respectively. The arrowheads in (**o**) indicates the clumps of cells in the proximal region of the muscle mass, while the asterisk represent the missing cells at the most distal part. All statistical comparison were performed using the two-sided non-parametric Wilcoxon test. NS: $p>0,05$. Box plots show the median (center line), 25th and 75th percentiles (box limits), and whiskers extending to the smallest and largest values within 1.5xIQR. Scale bars: 10µm (**a-d**,), 50µm (**g-l**) or 100µm (**n-o**).

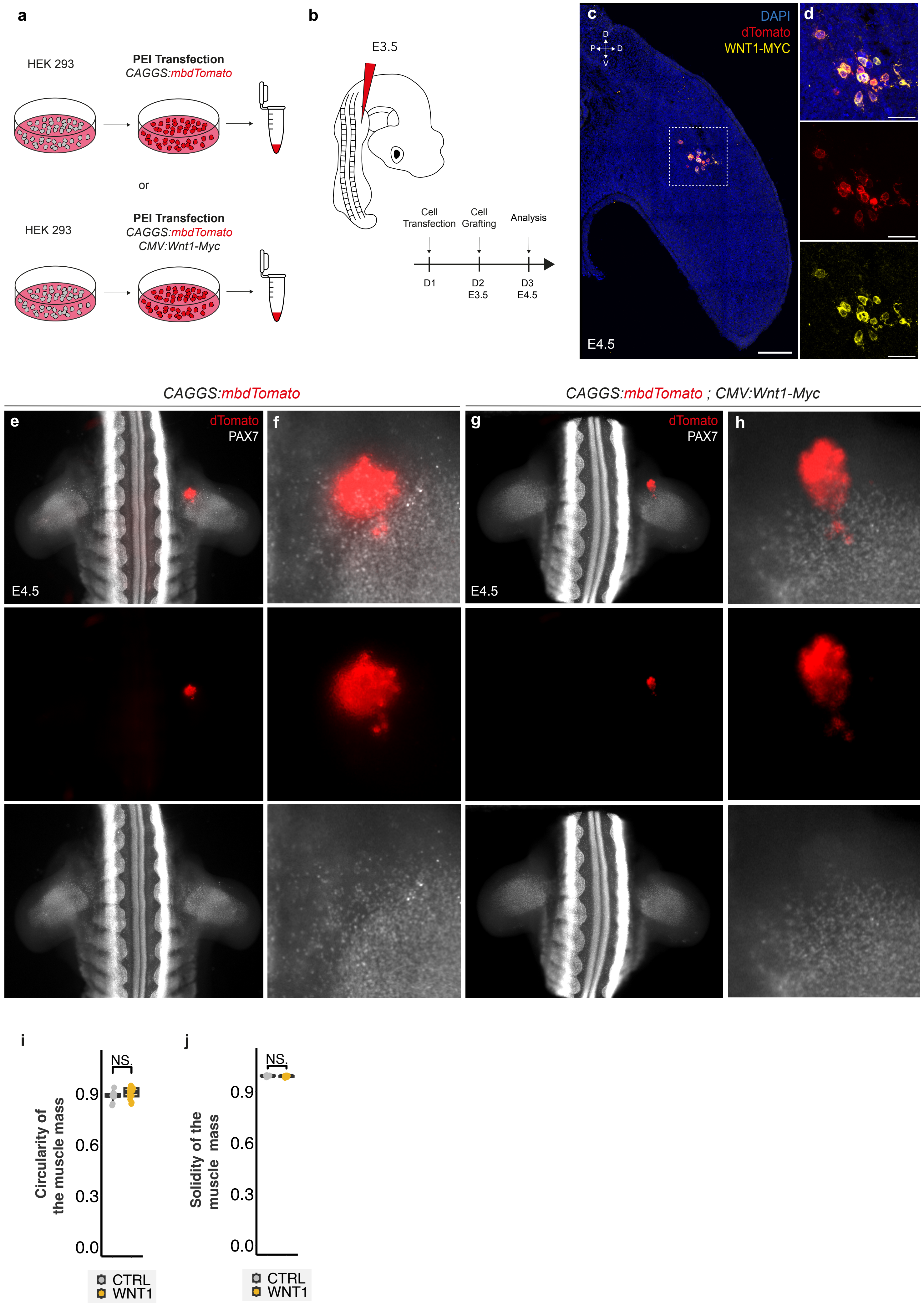
a
HEK 293
PEI Transfection
CAGGS:mbdTomato
or
HEK 293
PEI Transfection
CAGGS:mbdTomato
CMV:Wnt1-Myc
b
E3.5
Cell Transfection
Cell Grafting
Analysis
D1
D2
E3.5
D3
E4.5
c
D
P
D
V
DAPI
dTomato
WNT1-MYC
E4.5
d
CAGGS:mbdTomato
CAGGS:mbdTomato ; CMV:Wnt1-Myc
e
dTomato
PAX7
E4.5
f
g
dTomato
PAX7
E4.5
h
i
Circularity of the muscle mass
NS.
0.9
0.6
0.3
0.0
CTRL
WNT1
j
Solidity of the muscle mass
NS.
0.9
0.6
0.3
0.0
CTRL
WNT1

Supplementary Figure 10: Wnt does not play a trophic role on the migration of limb myoblasts. **a**,**b** HEK-293 cells were transfected with polyethylenimine (PEI) using either a plasmid coding for a ubiquitous membrane-bound dTomato in combination or not with a plasmid coding for a ubiquitous mouse Wnt1 in phase with a Myc tag. The day following the transfection cells were harvested and grafted in the anterior part of the growing limb bud of E3.5 chicken embryos. **c**,**d** Longitudinal section of a E4.5 limb grafted with Wnt1-expressing HEK-293 and immunostained for MYC. **e**-**h** Dorsal view of a E4.5 embryo after grafting of control HEK-293 (**e**,**f**) or with Wnt1-expressing HEK-293 (**g**,**h**) and immunostained for PAX7. **i**,**j** Quantification of the circularity and the solidity of the dorsal muscle mass following graft of control or Wnt1-expressing HEK-293, n=10 and 15, respectively. All statistical comparison were performed using the two-sided non-parametric Wilcoxon test. NS: $p>0,05$. Box plots show the median (center line), 25th and 75th percentiles (box limits), and whiskers extending to the smallest and largest values within 1.5xIQR. Scale bars: 100µm (**c**) or 20µm (**d**)

a

BD FACSDiva 9.0.1

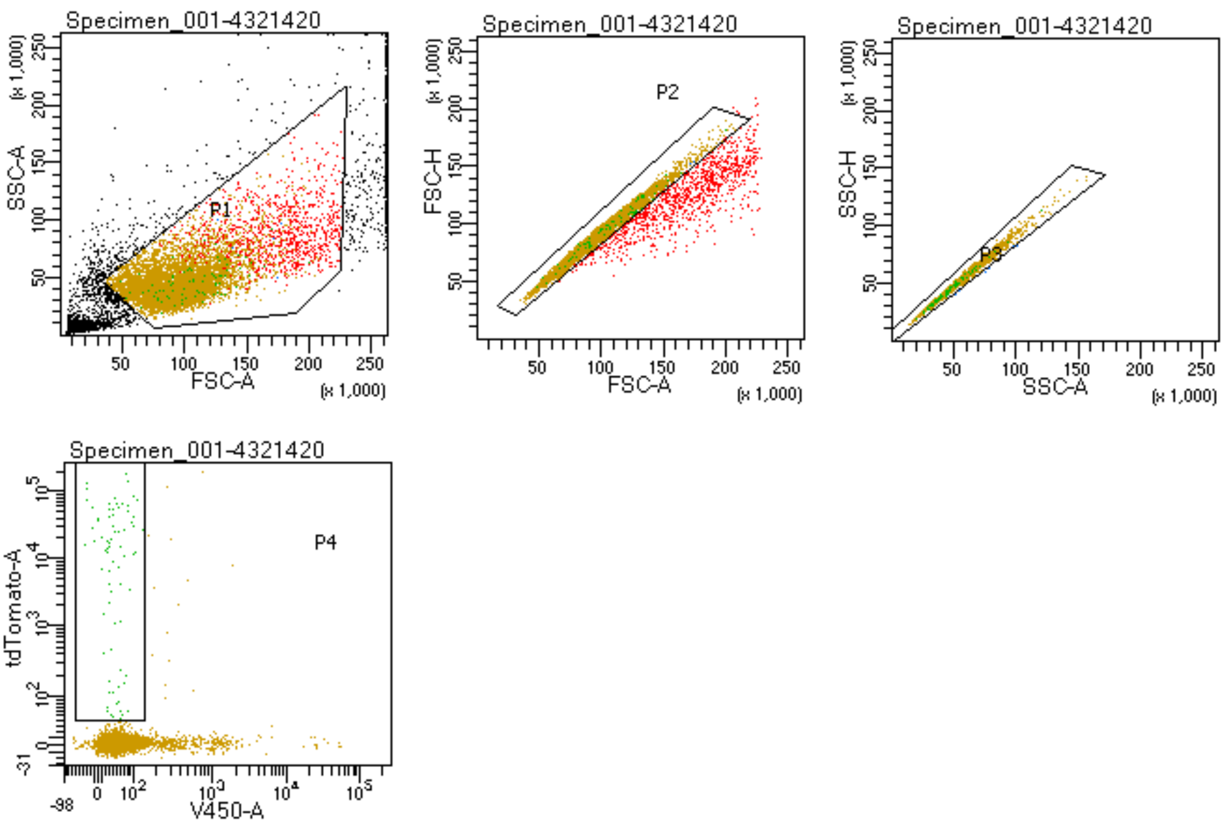

b

Tube: 4321420

Population	#Events	%Parent	%Total
All Events	10,000	####	100.0
P1	7,624	76.2	76.2
P2	6,592	86.5	65.9
P3	6,584	99.9	65.8
P4	77	1.2	0.8

Supplementary Figure 11: FACS gating and sorting workflow for the scRNA-seq. a Dead and live cell were sorted based on their Forward Scatter Area (FSC-A), Forward Scatter Height (FSC-H), Side Scatter Area (SSC-A) and Side Scatter Height (SSC-H) (P1, P2 and P3), as well as their DAPI incorporation (P4). **b** dTomato^{+} DAPI^{-} cells represented only 0,8% of all the events and 1,2% of all the live cells.

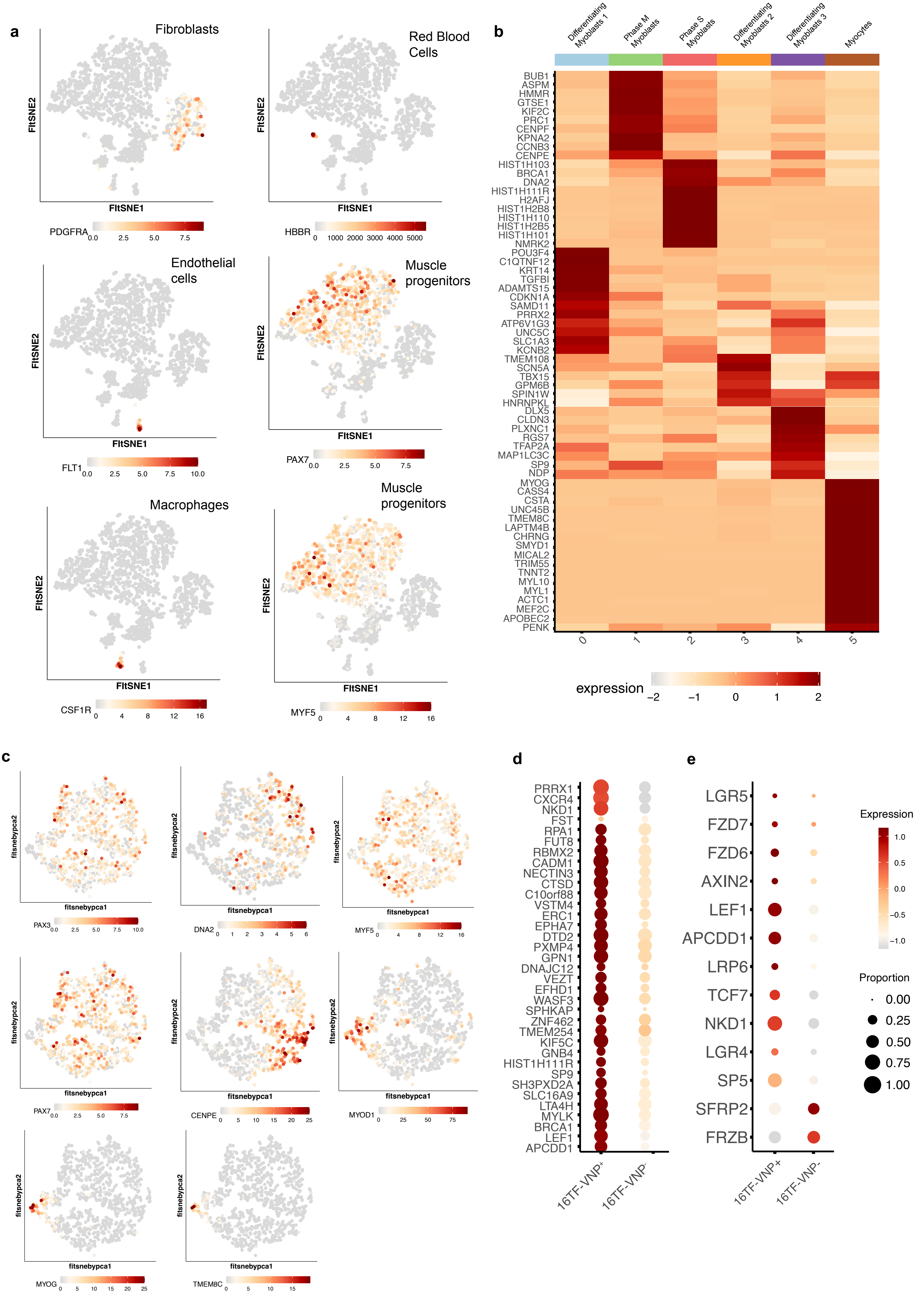

a
Fibroblasts
FltSNE2
FltSNE1
PDGFRA
0.0 2.5 5.0 7.5
Red Blood Cells
HBBR
0 1000 2000 3000 4000 5000
Endothelial cells
FLT1
0.0 2.5 5.0 7.5 10.0
Muscle progenitors
PAX7
0.0 2.5 5.0 7.5
Macrophages
CSF1R
0 4 8 12 16
Muscle progenitors
MYF5
0 4 8 12 16
b
Differentiating Myoblasts 1
Phase M Myoblasts
Phase S Myoblasts
Differentiating Myoblasts 2
Differentiating Myoblasts 3
Myocytes
BUB1
ASPM
HMMR
GTSE1
KIF2C
PRC1
CENPF
KPNA2
CCNB3
CENPE
HIST1H103
BRCA1
DNA2
HIST1H111R
H2AFJ
HIST1H2B8
HIST1H110
HIST1H2B5
HIST1H101
NMRK2
POU3F4
C1QTNF12
KRT14
TGFBI
ADAMTS15
CDKN1A
SAMD11
PRRX2
ATP6V1G3
UNC5C
SLC1A3
KCNB2
TMEM108
SCN5A
TBX15
GPM6B
SPIN1W
HNRNPKL
DLX5
CLDN3
PLXNC1
RGS7
TFAP2A
MAP1LC3C
SP9
NDP
MYOG
CASS4
CSTA
UNC45B
TMEM8C
LAPTM4B
CHRNG
SMYD1
MICAL2
TRIM55
TNNT2
MYL10
MYL1
ACTC1
MEF2C
APOBEC2
PENK
0 1 2 3 4 5
expression
−2 −1 0 1 2
c
fitsnebypca2
fitsnebypca1
PAX3
0.0 2.5 5.0 7.5 10.0
DNA2
0 1 2 3 4 5 6
MYF5
0 4 8 12 16
PAX7
0.0 2.5 5.0 7.5
CENPE
0 5 10 15 20 25
MYOD1
0 25 50 75
MYOG
0 5 10 15 20 25
TMEM8C
0 5 10 15
d
PRRX1
CXCR4
NKD1
FST
RPA1
FUT8
RBMX2
CADM1
NECTIN3
CTSD
C10orf88
VSTM4
ERC1
EPHA7
DTD2
PXMP4
GPN1
DNAJC12
VEZT
EFHD1
WASF3
SPHKAP
ZNF462
TMEM254
KIF5C
GNB4
HIST1H111R
SP9
SH3PXD2A
SLC16A9
LTA4H
MYLK
BRCA1
LEF1
APCDD1
16TF-VNP+
16TF-VNP-
e
LGR5
FZD7
FZD6
AXIN2
LEF1
APCDD1
LRP6
TCF7
NKD1
LGR4
SP5
SFRP2
FRZB
16TF-VNP+
16TF-VNP-
Expression
1.0
0.5
0.0
−0.5
−1.0
Proportion
0.00
0.25
0.50
0.75
1.00

Supplementary Figure 12: Single-cell transcriptomic analysis of sorted myoblasts. a Expression of various markers for different cell lineages in the raw dataset. **b** Heatmaps showing the expression of the most differentially expressed genes for each myogenic cluster. **c** Representation of some key genes involved in myogenesis. **d** Bubbleplot of the most differentially expressed genes in the 16TF-VNP^{+} population compared to the 16TF-VNP^{-}one. **e** Differential gene expression of Wnt-TCF/LEF related genes in the in the 16TF-VNP^{+} population compared to the 16TF-VNP^{-} one.

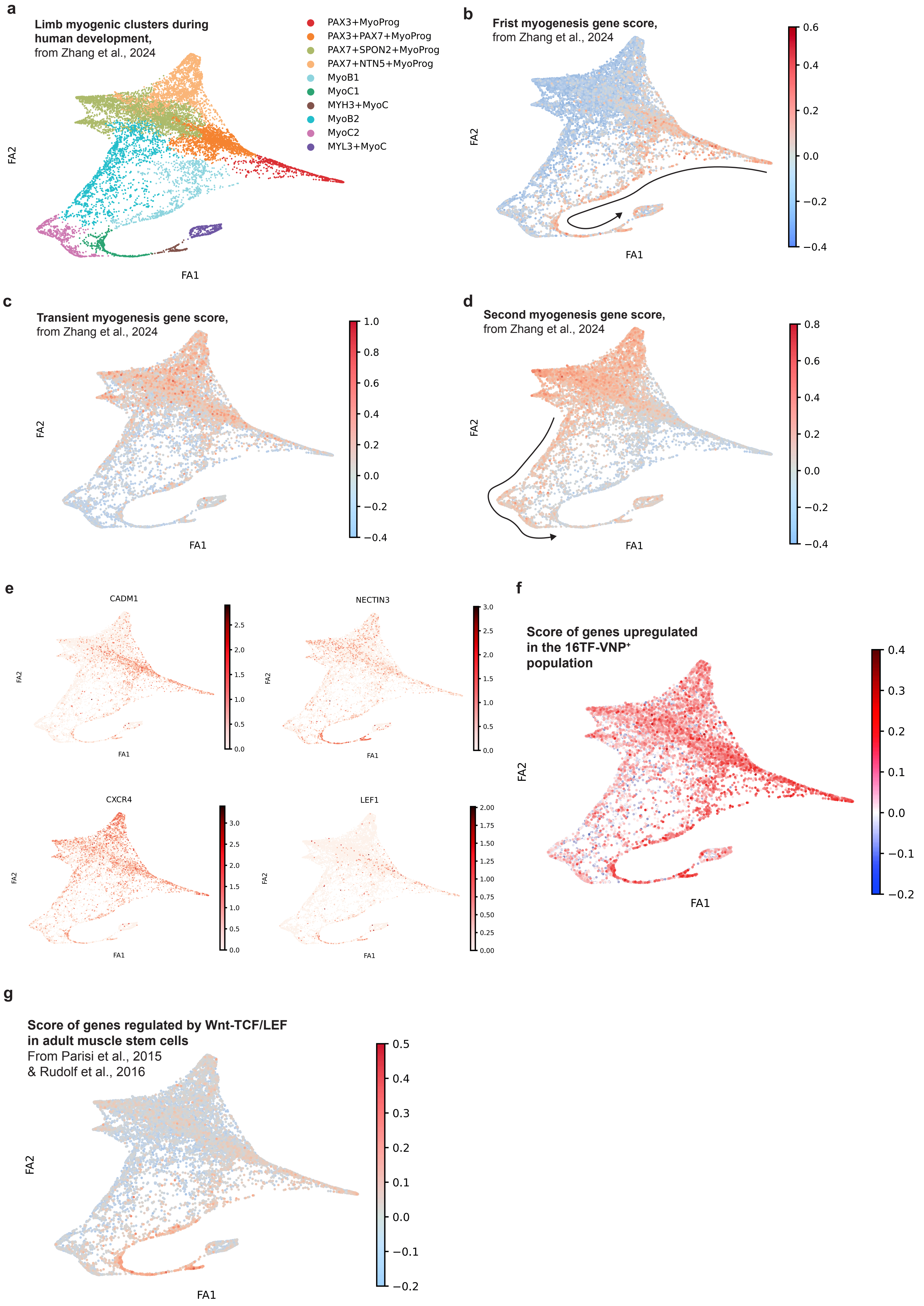
a
Limb myogenic clusters during human development,
from Zhang et al., 2024
PAX3+MyoProg
PAX3+PAX7+MyoProg
PAX7+SPON2+MyoProg
PAX7+NTN5+MyoProg
MyoB1
MyoC1
MYH3+MyoC
MyoB2
MyoC2
MYL3+MyoC
FA1
FA2
b
Frist myogenesis gene score,
from Zhang et al., 2024
FA1
FA2
c
Transient myogenesis gene score,
from Zhang et al., 2024
FA1
FA2
d
Second myogenesis gene score,
from Zhang et al., 2024
FA1
FA2
e
CADM1
NECTIN3
CXCR4
LEF1
f
Score of genes upregulated in the 16TF-VNP+ population
FA1
FA2
g
Score of genes regulated by Wnt-TCF/LEF in adult muscle stem cells
From Parisi et al., 2015
& Rudolf et al., 2016
FA1
FA2

Supplementary Figure 13: 16TF-VNP+ signature and Wnt-TCF/LEF signature are associated with the primary myogenesis during human limb development. **a** Force-directed (FA) graph layout of myogenic human cells during development, colored by cell clusters (from Zhang et al., 2024). **b-d** Force-directed (FA) graph layout of myogenic human cells during development, colored by scores of gene sets corresponding to the first (**b**), the transient (**c**) or the second (**d**) myogenesis identified by Zhang et al., 2024. **e** Expression of genes upregulated in the 16TF-VNP+ population in the human myogenic dataset. **f** Score of the genes upregulated in the 16TF-VNP+ population in the human myogenic dataset (see the materiel and methods section for a detailed description). **g** Score of known-genes regulated by Wnt-TCF/LEF signaling in adult muscle stem cells in the human myogenic dataset (see the materiel and methods section for a detailed description).
